# Supplementary material for: A whole-animal phenotypic drug screen identifies suppressors of atherogenic lipoproteins
Source: eLife. 2026 Aug 27;14:RP105314. doi: 10.7554/eLife.105314 (PMC13521566; doi:10.7554/eLife.105314)
Supplement: Supplementary file 2 [file elife-105314-code1.zip › Output_data/BIBIM_NL.pdf]

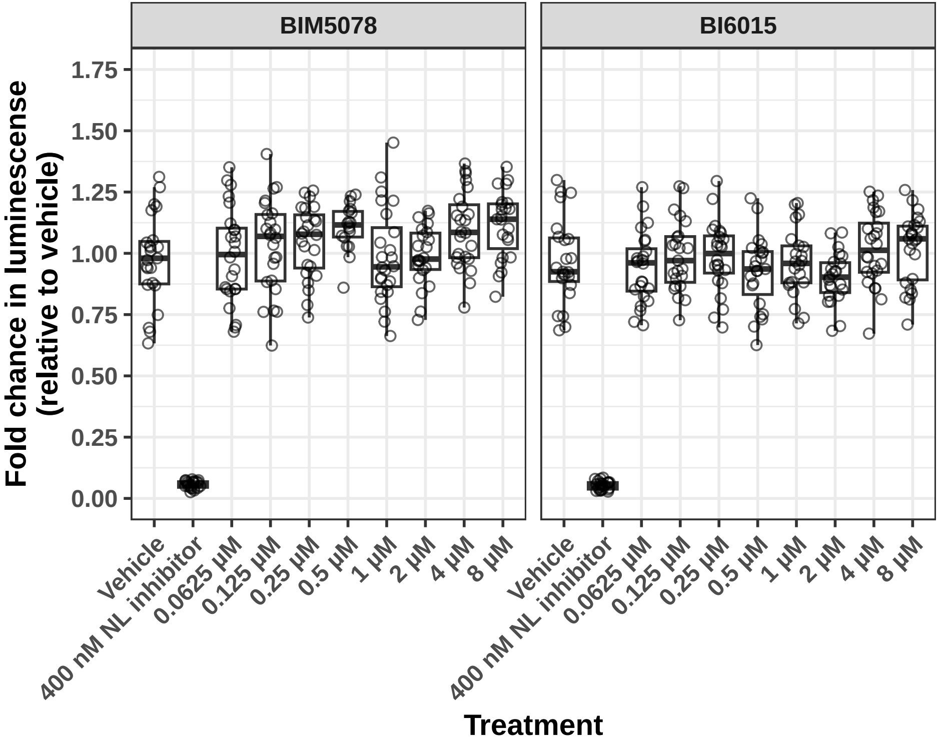

BIM5078
BI6015
Fold chance in luminescense
(relative to vehicle)
1.75
1.50
1.25
1.00
0.75
0.50
0.25
0.00
Vehicle
400 nM NL inhibitor
0.0625 μM
0.125 μM
0.25 μM
0.5 μM
1 μM
2 μM
4 μM
8 μM
Treatment
